# Melatonin Effects on Non-Alcoholic Fatty Liver Disease Are Related to MicroRNA-34a-5p/Sirt1 Axis and Autophagy

**DOI:** 10.3390/cells8091053

**Published:** 2019-09-08

**Authors:** Alessandra Stacchiotti, Ilaria Grossi, Raquel García-Gómez, Gaurangkumar Arvindbhai Patel, Alessandro Salvi, Antonio Lavazza, Giuseppina De Petro, Maria Monsalve, Rita Rezzani

**Affiliations:** 1 Division of Anatomy and Physiopathology, Department of Clinical and Experimental Sciences, University of Brescia, Viale Europa 11, 25123 Brescia, Italy; 2 Interdipartmental University Center of Research “Adaptation and Regeneration of Tissues and Organs (ARTO)”, University of Brescia, 25123 Brescia, Italy; 3 Division of Biology and Genetics, Department of Molecular and Translational Medicine, University of Brescia, 25123 Brescia, Italy (I.G.) (A.S.) (G.D.P.); 4 Instituto de Investigaciones Biomédicas “Alberto Sols” (CSIC-UAM), 28029 Madrid, Spain (R.G.-G.) (G.A.P.) (M.M.); 5 Instituto Zooprofilattico Sperimentale della Lombardia ed Emilia-Romagna (IZSLER), 25124 Brescia, Italy

**Keywords:** melatonin, nonalcoholic fatty liver disease, SIRT1, microRNA-34a-5p, obesity

## Abstract

Melatonin, an indole produced by pineal and extrapineal tissues, but also taken with a vegetarian diet, has strong anti-oxidant, anti-inflammatory and anti-obesogenic potentials. Non-alcoholic fatty liver disease (NAFLD) is the hepatic side of the metabolic syndrome. NAFLD is a still reversible phase but may evolve into steatohepatitis (NASH), cirrhosis and carcinoma. Currently, an effective therapy for blocking NAFLD staging is lacking. Silent information regulator 1 (SIRT1), a NAD+ dependent histone deacetylase, modulates the energetic metabolism in the liver. Micro-RNA-34a-5p, a direct inhibitor of SIRT1, is an emerging indicator of NAFLD grading. Thus, here we analyzed the effects of oral melatonin against NAFLD and underlying molecular mechanisms, focusing on steatosis, ER stress, mitochondrial shape and autophagy. Male C57BL/6J (WT) and SIRT1 heterozygous (HET) mice were placed either on a high-fat diet (58.4% energy from lard) (HFD) or on a standard maintenance diet (8.4% energy from lipids) for 16 weeks, drinking melatonin (10 mg/kg) or not. Indirect calorimetry, glucose tolerance, steatosis, inflammation, ER stress, mitochondrial changes, autophagy and microRNA-34a-5p expression were estimated. Melatonin improved hepatic metabolism and steatosis, influenced ER stress and mitochondrial shape, and promoted autophagy in WT HFD mice. Conversely, melatonin was ineffective in HET HFD mice, maintaining NASH changes. Indeed, autophagy was inconsistent in HET HFD or starved mice, as indicated by LC3II/LC3I ratio, p62/SQSTM1 and autophagosomes estimation. The beneficial role of melatonin in dietary induced NAFLD/NASH in mice was related to reduced expression of microRNA-34a-5p and sterol regulatory element-binding protein (SREBP1) but only in the presence of full SIRT1 availability.

## 1. Introduction

Non-alcoholic fatty liver disease (NAFLD) or fatty liver is the benign evidence of the metabolic syndrome, which affects 25% of the obese adult population worldwide. If unresolved, NAFLD evolves into steatohepatitis (NASH), cirrhosis and hepatocellular carcinoma (HCC) [1,2,3,4]. To date, there is not a peculiar therapy able to block the onset, progression and staging of the disease and preclinical experimental rodent NAFLD models are indispensable to discover novel therapeutic targets [5,6]. 

NAFLD pathogenesis, according to “the multiple hit hypothesis” is progressive and multifactorial, associated to multiple events like disrupted innate immunity, prolonged endoplasmic reticulum stress and defective autophagy [7,8,9,10,11]. Indeed, NAFLD is defined also a “mitochondrial disease”, because damaged mitochondria and sustained production of reactive oxygen species have been detected in the liver of obese and diabetic patients [12,13]. Unfortunately, the adverse progression from simply fatty liver to severe NASH and HCC is characterized by persistent mitochondrial damage [14]. Mitochondrial shape and turnover in the liver are strictly associated with their energetic function and the balance between fusion and fission processes is essential. Preclinical animal models of obesity indicated the prevalence of abnormal short mitochondria, an index of fission, unable to produce energy, so molecules that maintain mitochondrial shape are promising in treating obesity. The induction of selective cleaning and renovation of damaged mitochondria by lysosomes, called mitophagy, represents another promising therapeutic strategy in obesity and liver diseases [15,16].

Sirtuin 1 (SIRT1), the first member of mammal homologue of class III histone deacetylases, regulates longevity and cellular metabolism. In particular, SIRT1 potentiates fatty acid oxidation, mitochondrial biogenesis and turnover through deacetylation of its targets like peroxisome proliferator-activated receptor gamma coactivator-1-alpha (PGC-1alpha) [17,18,19]. SIRT1 up-regulation is effective against obesity and insulin resistance in NAFLD rodents [20]. Interestingly, SIRT1 limits overweight downregulating the sterol response element-binding protein (SREBP-1c), a key activator of de novo lipogenesis in the liver and activating β-oxidation in hepatocytes [21,22]. By contrast, SIRT1 down-regulation or full silencing accentuate fatty liver and inflammation [23]. Moreover, low plasmatic and hepatic SIRT1 levels have been found in obese and NAFLD patients [24,25]. 

Growing evidences indicate that microRNAs (miRNAs), small non-coding RNAs (19-22 nucleotides in length), can act as negative regulators of SIRT1 gene expression by controlling the translation and stability of the mRNA [26,27]. Yamakuchi et al. demonstrated that microRNA-34a-5p (miR-34a-5p) inhibited SIRT1 expression through a direct miR-34a-binding site within the 3′UTR of SIRT1 mRNA [28]. Intriguingly, elevated miR-34a-5p levels have been measured in the plasma and in the liver of obese patients and dietary obese and leptin-deficient mice in NAFLD and NASH models [29,30]. Moreover, miR-34a-5p, via SIRT1 inhibition, induces ER stress and inflammation in the liver of rodents and humans [31,32].

Many studies indicated that melatonin (N-acetyl-5-methoxytryptamine), the nighttime indole produced by pineal and many extra-pineal tissues, is effective against the metabolic syndrome [33,34,35]. Recently, melatonin reverses harmful effect of dietary fructose in animal models modulating metabolic pathways like lipogenesis and lipolysis, beta-oxidation, gluconeogenesis [36]. Lipophilic melatonin freely passes through all biological membranes and accumulates mainly into mitochondria where influences mitochondrial structure and function via PGC1 alpha signal [37,38]. Melatonin may be taken in the liver also by specific cellular and nuclear receptors in a dose-dependent manner [39]. 

We previously reported that melatonin attenuated NAFLD in leptin-deficient mice and in hypercholesteremic Apo E mice by enhancing SIRT1 protein in hepatocytes [40,41]. The finding is not surprising because there is a strict relationship between these two molecules in the regulation of circadian rhythms and metabolism in animal models and cells [42]. 

Nevertheless, dietary induced NAFLD in rodents has a greater translational value, because nutrients overload is the main cause of hepatic metabolic syndrome in humans [43]. However, rodent NAFLD/NASH models are heterogeneous and dependent on the duration of the treatment, dietary fat composition, strain or gender and microbiota [44,45,46].

Considering that the effect of melatonin in dietary induced NAFLD in animals partially deprived of SIRT1 expression is still unknown, we carried out this study. Male C57BL/6J mice (WT) and heterozygous SIRT1+/− mice (HET) were placed on a high fat diet (HFD) or on standard maintenance diet (STD), drinking or not drinking melatonin at 10 mg/kg for 16 weeks from 12 to 28 weeks of age. We have chosen a relatively short time of overfeeding because we are interested in the study of the appearance and early phases of liver damage and not a full-blown condition [47]. 

Thus, we evaluated whether melatonin could attenuate steatosis, ER stress, lipid peroxidation, inflammation, mitochondrial damage and autophagy in hepatocytes in WT and HET mice. 

Finally, to demonstrate whether melatonin benefit could correlate with the inverse miR-34a/SIRT1 regulation, we analyzed miR-34a-5p and SIRT1 expression in all experimental groups. 

## 2. Materials and Methods

### 2.1. Animal Model and Treatments

SIRT1+/− TG mice on the 129/J background were kindly provided by Frederick W. Alt, Harvard University Medical School, Boston, MA. The animals were back-crossed with C57BL/6 mice for five generations to obtain heterozygous mice (HET) with the C57BL/6 background. Male HET and wild type (WT) littermates were used in this study. The mice were housed in a temperature and humidity controlled-facility with a 12 h/12 h light–dark cycle and free access to standard rodent diet (8.4 E% lipids, 72.4 E% carbohydrates, 19.3 E% proteins; A0 Safe Laboratories, Augy, France) and water up to 12 weeks of age. After that, mice were maintained on standard diet (STD) or placed on a high-fat diet (HFD; 58.4 E% lard, 26.6 E% carbohydrates, 15 E% protein; Envigo TD03584) for further 16 weeks. At the same time, some animals drank melatonin (a kind gift from Flamma Spa, Italy) at a dose of 10 mg/kg of body weight. The duration of the treatment was chosen according to Xu et al. and melatonin dosage according to previously described [23,48]. All treatments were carried out in compliance with the European Community Commission directive guidelines (86/609/EEC) and approved by local CSIC Ethical Committee (code D.N.I. 50840973W). Animals (*n* = 6–10/ group) were randomly assigned to the following groups: WT mice fed maintenance diet, drinking melatonin or not; HET mice fed maintenance diet, drinking melatonin or not; WT mice fed an HFD drinking melatonin or not; HET mice fed an HFD drinking melatonin or not. All experiments were performed under controlled conditions and animals have free access to diets and water. Body weight was assessed weekly. Euthanasias were performed during the light phase in the morning starting from 10:00 onwards.

### 2.2. Indirect Calorimetry and Glucose Tolerance Test

Indirect calorimetry was performed using a Phenomaster system (TSE Systems, Bad Homburg, Germany). WT and HET mice were acclimatized in metabolic chambers 3 days before the beginning of the study. Mice (*n* = 8/group) were placed on HFD or HFD plus melatonin for 16 weeks. Indirect calorimetry data were recorded at the beginning (T0), 7 (T7) or 15 (T15) weeks of high fat diet. The following parameters were determined: food intake (Feed), water intake (Drink), motor activity (XT + YT), oxygen consumption (VO2), CO_2_ production (VCO2), respiratory exchange ratio (RER) and energy expenditure (H1). The data were collected for five continuous days but only data from the three days in the middle were chosen for analysis. 

Intraperitoneal glucose tolerance test (IP-GTT) was performed on fasted mice (*n* = 5/group) by a single intraperitoneal glucose injection at 2 g/kg. The blood glucose levels were measured before starting (*t* = 0) and 15, 30, 60 and 120 min (*t* = 15, *t* = 30, *t* = 0 and *t* = 120) after injection by tail bleed. The data are expressed as mg/dL.

### 2.3. Histopathology 

At the end of the treatments, the livers were removed, fixed in buffered formalin for 24 h and embedded in paraffin for histology and immunohistochemistry. Hematoxylin and eosin staining were used for NAFLD activity score evaluation (NAS), PicroSirius Red and Azan trichrome for fibrosis and Perls for iron deposition. Sirius red positive areas of Zones 1 and 3 were estimated as previously [41]. 

### 2.4. Immunohistochemistry 

Briefly, liver sections (4 µm thick) were subjected to antigen retrieval and block of endogenous peroxidase activity and avidin-biotin-peroxidase method according to previous study [40]. The slides were incubated with normal serum from species producing the secondary antibody and subsequently with primary polyclonal antibodies against 4HNE (1:400, Abcam, #ab46545, Cambridge, UK), SIRT1 (1:150, Santa Cruz Biotechnology, #sc15404, Dallas, TX, USA), GRP78 (1:300, Abcam, #ab21685), SREBP1 (1:100, Santa Cruz Biotechnology, #sc8984), IL6 (1:100, Santa Cruz Biotechnology, #sc1265), p62/SQSTM1 (1:50, MBL International, Woburn, MA, USA) or monoclonal antibodies against F4/80 (1:50, Bio Rad, #MCA497GA, Segrate, Italy), Mitofusin 2 (1:200, Abnova, #H00009927, Taipei, Taiwan). All experiments were performed in triplicate. The staining intensity was expressed as arbitrary units (AU) or percentage of positive nuclei, in 20 randomly chosen microscopic fields, using an image analyzer (Image Pro Premier 9.1, Media Cybernetics, Rockville, MD, USA).

### 2.5. Analysis of the Autophagy In Vivo

WT and HET mice (*n* = 6) were fasted for 24 h then fed a STD for 2 h (from 22:00 to 24:00) in the dark and starved again for an additional 24 h. To block autophagy and measure late autophagosomes, other WT and HET mice (*n* = 6) were intraperitoneally injected with leupeptin, a lysosomal inhibitor, at a dose of 15 mg/kg body weight 1 h prior to euthanasia [49]. 

### 2.6. Transmission Electron Microscopy 

Liver fragments were fixed in 2.5% glutaraldehyde, post-fixed in 2% osmium tetroxide in cacodylate buffer and embedded in Epon as we previously described [40]. Lipid droplets area was estimated on semithin sections at 1000× magnification and deeply at 5000× using ultrastructural stereology in pericentral hepatocytes [50]. Mitochondria parameters were measured under a transmission electron microscope (TEM-Tecnai G2 Spirit, FEI, Eindhoven, The Netherlands) according to a previous study [40]. Late autophagosomes, as single membrane-vesicles containing an electron-dense content, were estimated on 20 electron micrographs randomly taken at 45,000× [51]. 

### 2.7. RNA Isolation and Quantitative Real-Time PCR for miRNA and Gene Expression 

The total RNAs, including small RNAs, were isolated from the liver of each mouse (three mice for each condition) using the miRNeasy Mini Kit (Qiagen, Hilden, Germany), according to the manufacturer’s instructions. Mature miR-34a-5p was quantitated through TaqMan Advanced miRNA Assays, using pre-formulated primers and probes obtained from Applied Biosystems (Foster City, CA, USA). The quantitative PCR (qPCR) reaction was performed in 20 μL containing 5 μL of cDNA template, TaqMan Fast Advanced Master Mix (2×) and TaqMan Advanced miRNA Assay (20×) specific for miR-34a-5p (ID Mmu481304) or let-7g-5p as an internal control (ID Mmu478580). The PCR mixtures were incubated at 95 °C for 20 s, followed by 40 cycles at 95 °C for 3 s and 60 °C for 30 s. The SIRT1 and SREBP1 mRNA expression levels were evaluated using the TaqMan Gene Expression Assay, as previously described [52]. qPCR reaction was performed in triplicate for each sample using the 7500 real time PCR system. The relative quantification (RQ) of miR-34a-5p and SIRT1 expression was calculated using 2^–ΔΔCt^ method, where ΔΔC_t_ = (Ct_target_ − Ct_reference_) _SAMPLE_ – (Ct_target_ − Ct_reference_) _CALIBRATOR_. The expression of let-7g-5p and GAPDH was used to normalize miR-34a-5p and SIRT1 levels, respectively. The sample with the lowest expression level of target gene was defined as calibrator [53,54].

### 2.8. Western Blot Analysis

Liver homogenates were obtained by NE-PER nuclear and cytoplasmic extraction reagents (Thermo Fisher Scientific, Waltham, MA, USA). Constant amounts of protein were loaded on 10% SDS-polyacrylamide gels and electro-transferred on nitrocellulose membranes. The following primary antibodies were used: anti-mouse LC3B (1:1000 in 1% BSA, Cell Signalling Technology, #D11, Leiden, The Netherlands), anti-mouse SIRT1 (1:1000, Abcam, ab110304. Cambridge, UK), anti-mouse β-actin (1:2000, Santa Cruz Biotechnology, sc8432), anti-mouse α-tubulin (1:2000, Abcam, #ab4074), anti-GRP78 (1:1000, Abcam, ab21685), anti-rabbit p62/SQSTM1 (1:1000, MBL International, Woburn, MA, USA). Primary antibodies were stained using peroxidase-conjugated secondary anti-rabbit or anti-mouse IgG (1:5000, Vector Laboratories, Burlingame, CA, USA). The immunoreaction was detected with 3, 3′-diaminobenzidine tetrahydrochloride for peroxidase stain. Densitometric analysis of bands was performed using the Gel-Pro Analyzer software (4.5, Media Cybernetics Inc., Silver, MD, USA).

### 2.9. Statistical Analysis

Statistical analyses were performed by two-way analysis of variance (ANOVA) using Graph Pad Prism 6 software (6.02.328, San Diego, CA, USA). The Bonferroni test was used to determine differences between single treatment groups. Data expressed as mean ± SEM and *p*-values <0.05 were considered significant. Glucose tolerance test values were analyzed by Student’s *t*-test.

## 3. Results

### 3.1. Melatonin Improved Metabolism and Lipid Peroxidation in WT but Not in HET Mice Fed a High-Fat Diet

**Figure 1:**
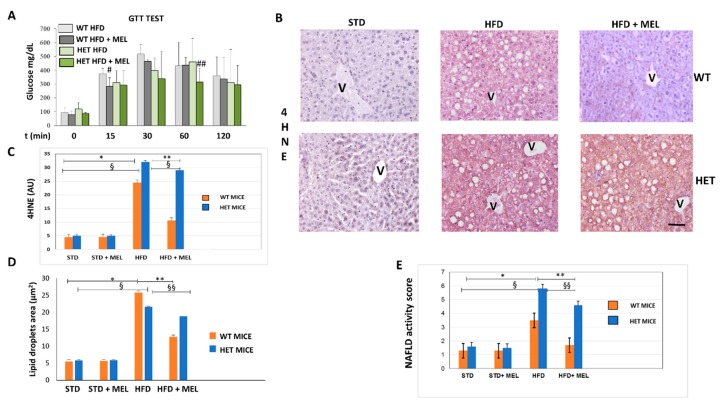
Melatonin attenuated glycaemia, lipid peroxidation and steatosis in the liver of wild type (WT) mice, not in SIRT1 heterozygous (HET) mice placed on a high-fat diet. (**A**) Glucose transport test (GTT) measured blood glucose in WT and HET mice fed a maintenance (STD) or high-fat diet (HFD) for 16 weeks. # *p* < 0.05 vs. WT HFD at 15 min; ## *p* < 0.05 vs. HET HFD at 60 min. (**B**) Representative pictures of 4-hydroxynonenal (4HNE) immunostaining labeled as brown color drop. Intense lobular signal was evident in hepatocytes in HFD mice but melatonin reduced 4HNE staining in WT, not in HET. (**C**) The quantification of 4HNE immunohistochemistry. (**D**) Lipid droplets area estimated on semithin sections. (**E**) Non-alcoholic fatty liver disease (NAFLD) activity score an index of liver damage. STD: standard rodent diet; HFD: high-fat diet; HFD + MEL: high fat diet plus melatonin; V: central vein; scale bars indicate 10 µm in A and B. Values are mean ± SEM. * *p* < 0.05 vs. WT STD, ** *p* < 0.05 vs. WT HFD, § *p* < 0.05 vs. HET STD, §§ *p* < 0.05 vs. HET HFD.

Indirect calorimetry in WT and HET mice placed on a high fat diet (HFD) drinking or not drinking melatonin was performed at the beginning of treatments, after 7 or 15 weeks of dietary intake. WT HFD mice with oral melatonin supplementation maintained significantly higher O_2_ consumption and CO_2_ production rates than HET mice after 15 weeks of diet (Figure S1A,B). Moreover, energy expenditure was lower in HET mice despite drinking melatonin (Figure S1D). Whereas all groups of animals (Figure S1E) have taken on the same amount of food and water, WT mice, drinking melatonin, probably dissipated the energy provided by fat better than HET mice. This interesting trend was particularly evident during the hours of dark, when melatonin compensated for the HFD induced loss in locomotor activity largely in WT rather than HET mice (Figure S1F). To evaluate the impact of diets on glucose homeostasis, we performed a blood glucose tolerance test. HFD induced glucose intolerance in WT and HET mice, with glucose homeostasis being slightly impaired in SIRT1 HET mice at baseline. However, melatonin improved glucose tolerance during the early fast response phase (at 15 min following glucose administration) in WT (p = 0.0971) and at late phase (at 60 min following glucose administration) in HET mice (p = 0.0972) (Figure 1A). Expression of 4-hydroxynonenal (4HNE), a lipid peroxidation marker, negligible in maintenance diet fed mice (STD), was moderate in WT HFD and strong in HET HFD mice (Figure 1B). Melatonin attenuated its signal in WT HFD mice but was ineffective in HET HFD mice. Densitometry is summarized in Figure 1C.

### 3.2. Melatonin Attenuated Steatosis and ER Stress in WT but Not in HET Mice Placed on a High-Fat Diet

**Figure 2:**
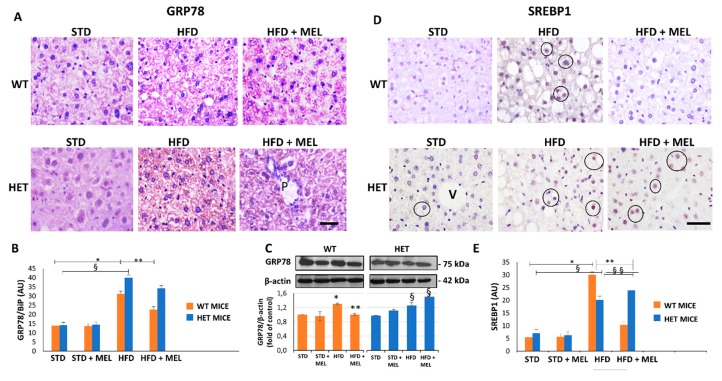
Melatonin reduced endoplasmic reticulum stress and lipogenesis in wild type (WT) mice, not in SIRT1 heterozygous (HET) mice on a high-fat diet. (**A**) Representative pictures of GRP78 protein staining in the liver. (**B**) Densitometric analysis of GRP78 staining. (**C**) GRP78 Western blotting and relative quantification. (**D**) SREBP1 protein staining in WT and HET mice fed with a maintenance (STD) or high-fat diet. (HFD) with or without melatonin intake. (**E**) Densitometric analysis of SREBP1 staining. Values are mean ± SEM. Scale bars represent 10 µm. P: portal space; V: central vein. Dark circle: SREBP1-positive nuclei. * *p* < 0.05 vs. WT STD, § *p* < 0.05 vs. HET STD, ** *p* < 0.05 vs. WT HFD.

Light microscopy and TEM stereology indicated that after melatonin intake the lipid droplet volume decreased in WT HFD mice (Figure 1D and Figure S2A,B). Conversely, micro-steatosis persisted in HET HFD mice despite melatonin (Figure S2A). Given that endoplasmic reticulum stress influenced steatosis, we analyzed the expression of glucose regulated protein 78 kDa (GRP78), the master ER chaperone. GRP78 immunostaining was faint in controls, enhanced in WT and HET HFD mice, but decreased only in WT HFD mice drinking melatonin (Figure 2A,B). Conversely, in HET HFD mice a strong GRP78 reaction, as a sign of prolonged ER stress, and steatosis persisted despite melatonin. Western blotting experiments revealed a similar trend of GRP78 expression (Figure 2C). We also checked and quantified SREBP1 mRNA levels and expression by qPCR and by immunostaining. SREBP1 mRNA levels, elevated in WT HFD, were significantly reduced by melatonin. Interestingly, in HET HFD mice drinking melatonin they greatly enhanced (Figure S2C). SREBP1-positive nuclei were scarce in controls, then increased in WT and HET HFD groups, but decreased only in WT HFD plus melatonin (Figure 2D). Conversely, in HET HFD mice plus melatonin, SREBP1-positive nuclei increased (Figure 2E). These results indicate an abnormal lipogenesis in mice lacking proper SIRT1 expression.

### 3.3. Melatonin Was Ineffective against Inflammation and Fibrosis in HET Mice Fed a High-Fat Diet

**Figure 3:**
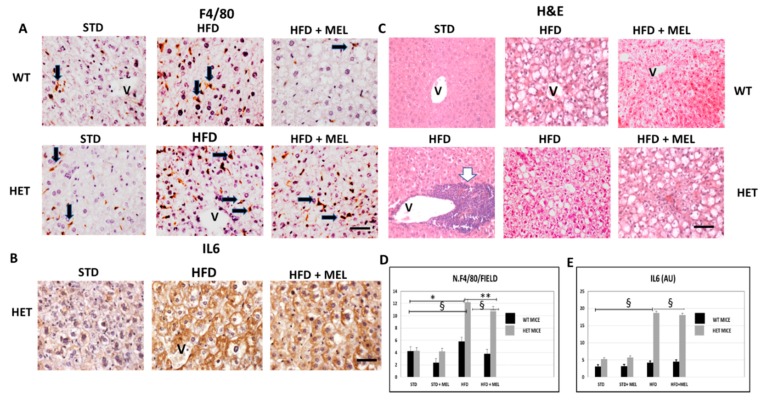
Melatonin maintained inflammation and steatosis in the liver of SIRT1 heterozygous (HET) mice on a high-fat diet for 16 weeks. (**A**) Representative pictures of F4/80 immunostaining in liver sections and (**D**) its quantification. (**B**) IL6 protein staining in the liver and (**E**) its quantification. (**C**) Representative pictures of hematoxylin and eosin staining of the liver. Values are mean ± SEM. Scale bars represent 10 µm. Black arrow: F4-positive cells. White arrow: large inflammatory aggregate. V: central vein. * *p* < 0.05 vs. WT STD, ** *p* < 0.05 vs. WT HFD, § *p* < 0.05 vs. HET STD.

After 16 weeks of HFD intake, fibrosis was absent in the liver of WT mice with or without melatonin (Figure S3A). In contrast, in HET HFD mice, a thick collagen deposition was evident in the peri-portal area (Figure S3A,D) and persisted in mice drinking melatonin (Figure S3B). To best characterize activated Kupffer cells, we performed Perls’ iron staining and F4/80 immunostaining. Interestingly, there was limited macrophage activation in WT HFD mice, but, in contrast, blue macrophages were present in HET HFD mice (Figure S3C,E). F4/80 immunostaining showed a similar trend in macrophages in HET HFD mice, despite melatonin intake (Figure 3A,D). Due to the crucial role of pro-inflammatory cytokines in NAFLD staging, we studied the interleukin 6 (IL6) in HET HFD mice. Remarkably, IL6 staining was negligible in WT HFD but evident in HET HFD mice drinking or not drinking melatonin (Figure 3B,E). Taken together, these data concurred to define the NAFLD activity score (NAS) [55] reported in Figure 1E. In WT HFD mice, the NAS score was above 3, but it significantly decreased after melatonin intake. In contrast, in HET HFD mice, NAS exceeded 6, the highest value, but was slightly decreased by melatonin intake. A NAS score higher than 5 is generally associated with a NASH stage in the liver. Representative pictures stained by hematoxylin and eosin have been included in Figure 3C.

### 3.4. Autophagic Flux Was Sustained in WT Mice but Not in HET Mice during Starvation

**Figure 4:**
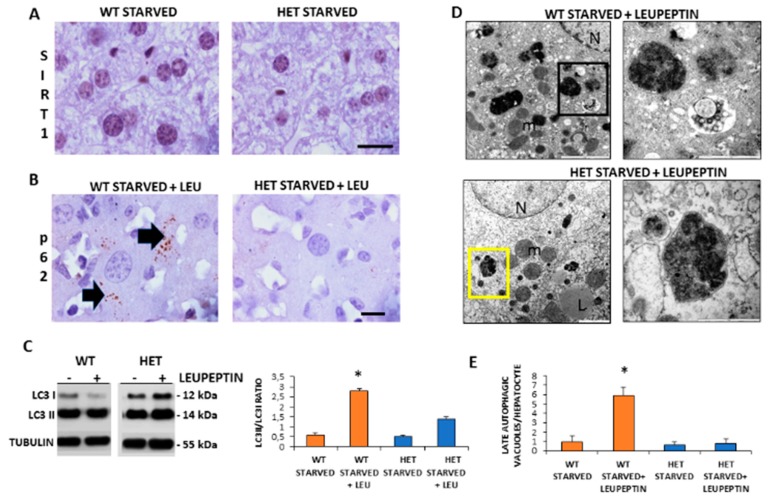
Autophagic flux is sustained in wild type (WT) but inconsistent in SIRT1 heterozygous (HET) mice liver during starvation. (**A**) Representative Sirtuin 1 protein staining in WT and HET mice. (**B**) Representative pictures of P62 protein dot staining (arrow) in starved mice plus leupeptin. (**C**) LC3I and LC3II expressions by Western blotting and relative quantification. (**D**) Representative transmission electron microscopic images of the liver in starved WT and HET mice injected with leupeptin, a lysosomal inhibitor. Black square, late autophagosome. Yellow square: lysosomes; m: mitochondria; N: nucleus; L: lipid droplet. (**E**) Quantification of late autophagosomes in the hepatocytes. Scale bars in immunohistochemistry represent 10 µm. Values are mean ± SEM. * *p* < 0.05 vs. WT starved.

Given that starvation triggered autophagy in the liver and SIRT1 enhanced in caloric restriction, we decided to investigate the SIRT1 expression and autophagic flux during starvation [9]. There were numerous brown SIRT1-immunopositive nuclei in starved WT mice, compared to STD WT mice and, in contrast, there were only scarce SIRT1 positive nuclei in starved HET mice (Figure 4A).

To measure the basal autophagic flux, we compared changes in the LC3II/LC3I ratio with or without leupeptin. Furthermore, we conducted p62/SQSTM1 immunostaining, hereinafter referred to simply as p62. A sustained LC3II/LC3I ratio and several p62 positive dots were observed in starved WT mice plus leupeptin compared to mice without leupeptin (Figure 4B). In contrast, in starved HET mice, the p62 immunostaining was negligible and the LC3II/LC3I ratio limited despite leupeptin (Figure 4C). To further characterize autophagy in the liver, we quantify autophagosomes by ultrastructural analysis. Numerous autophagic vacuoles, blunted by leupeptin, were detected only in starved WT mice (Figure 4D). Conversely, only primary lysosomes were present in HET mice (Figure 4D). The quantitative analysis confirmed these observations (Figure 4E). These data indicate that there was an active basal autophagy only in WT mice and not in HET mice.

### 3.5. Melatonin Triggered Autophagy and Mitophagy only in WT Mice Fed a High-Fat Diet

**Figure 5:**
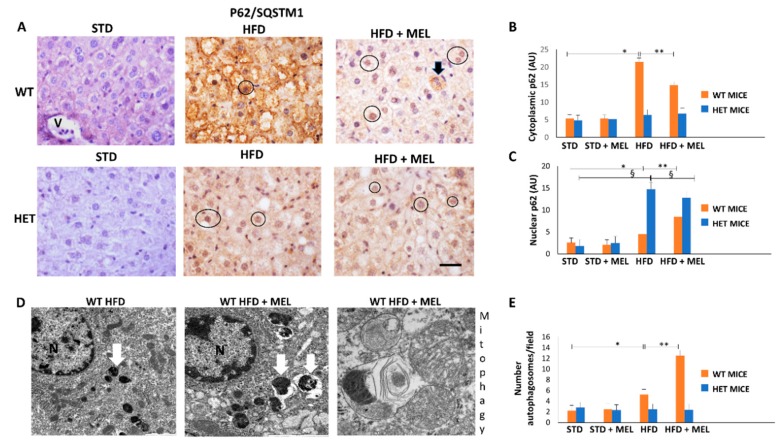
Melatonin recovered autophagy in the liver of wild type (WT) but not in SIRT1 heterozygous (HET) mice on a high-fat diet. (**A**) Representative pictures of P62/SQSTM1 immunohistochemistry in the liver. (**B**,**C**) Densitometric analysis of cytoplasmic and nuclear P62/SQSTM1 immunostaining. (**D**) Representative transmission electron micrographs of WT mice liver plus melatonin, indicating autophagy and mitophagy. (**E**) The quantification of autophagosomes. Scale bars in immunohistochemistry represent 10 µm. Black circle, P62-positive nucleus, White arrow: late autophagosome. Values are mean ± SEM. * *p* < 0.05 vs. WT STD, § *p* < 0.05 vs. HET STD, ** *p* < 0.05 vs. WT HFD.

Cytoplasmic p62 staining has been correlated to autophagy or mitophagy [56], but nuclear p62 to proliferative oncogenic transformation in the liver. In WT HFD mice, cytoplasmic p62 immunoreaction was intense but attenuated by melatonin intake (Figure 5A,B). In contrast, p62 signal was weak in HET mice under all experimental conditions. Western blotting was also performed and revealed a similar trend of expression (Figure S4). Notably, the nuclear p62 signal was limited in WT HFD mice but enhanced in mice when drinking melatonin. Conversely, the nuclear p62 positivity was strong in HET HFD mice and scarcely limited by melatonin (Figure 5C). All these data indicate that melatonin probably stimulates beneficial liver regeneration in WT mice but maintains pro-oncogenic changes already evident in HET HFD mice.

Using transmission electron microscopy, we were unable to observe autophagic vacuoles in hepatocytes in CTR and HET HFD mice (data not shown). By contrast, autophagosomes were evident in WT HFD mice and increased in mice drinking melatonin (Figure 5D,E). Interestingly, in WT HFD plus melatonin, we observed a late autophagic vacuole encircling mitochondria debris, which is a clear sign of mitophagy (Figure 5D). Thus, melatonin is a positive regulator of autophagy and probably mitophagy, but only in WT HFD mice livers.

### 3.6. Melatonin Recovered Mitochondria in WT Mice but Not in HET Mice Placed on a High-Fat Diet

**Figure 6:**
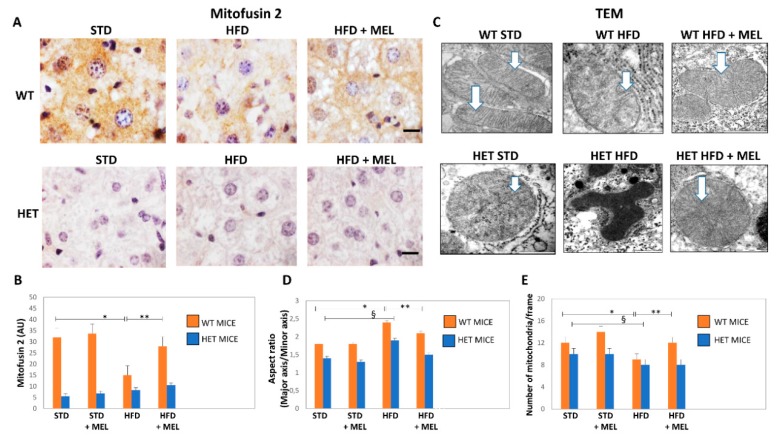
Melatonin produced elongated mitochondria and increased their number in wild type (WT) but not in SIRT1 heterozygous (HET) mice on a high-fat diet. (**A**) Representative pictures of Mitofusin 2 protein staining in the liver. (**B**) Densitometric analysis of Mitofusin 2 intensity. (**C**) Representative transmission electron micrographs of mitochondria. (**D**) Aspect ratio of mitochondria. (**E**) The number of mitochondria. White arrow: mitochondrial cristae. Scale bars in immunohistochemistry represent 10 µm. Values are mean ± SEM. * *p* < 0.05 vs. WT STD, § *p* < 0.05 vs. HET STD, ** *p* < 0.05 vs. WT HFD.

We explored the presence of elongated fused mitochondria by mitofusin 2 (Mfn2) immunostaining. In WT STD mice, Mfn2 immunostaining was intense, but it was weaker in HET STD mice (Figure 6A,B). However, Mfn2 immunostaining decreased in WT HFD mice and was partially restored by melatonin (Figure 6A,B). Conversely, a negligible Mfn2 signal was detected in all HET mice groups. These findings agree with mitochondria ultrastructure and morphometry that indicated the presence of mega-mitochondria with reduced cristae in WT HFD, then restored by melatonin (Figure 6C,E). By contrast, deformed or round mitochondria with short cristae were observed in HET mice, despite melatonin intake (Figure 6C,D).

### 3.7. Melatonin Upregulated SIRT1 and Downregulated miR-34a-5p in WT Mice Fed a High-Fat Diet

**Figure 7:**
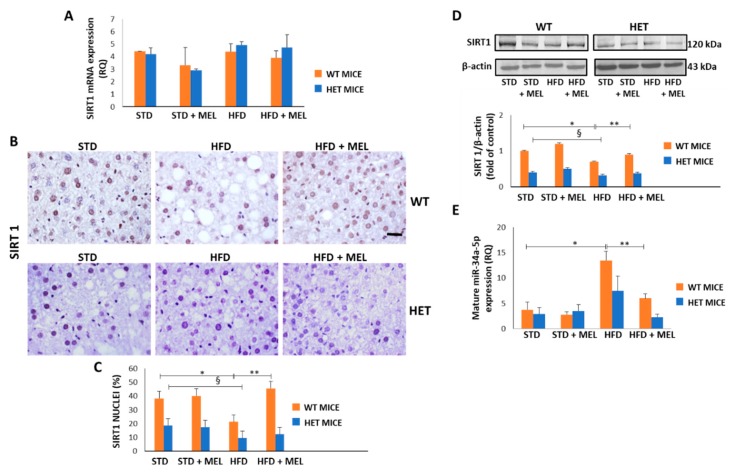
Melatonin restored Sirtuin 1 protein and modulated miR-34a-5p in the liver of wild type (WT) but not in heterozygous (HET) mice on a high-fat diet. (**A**) Sirtuin 1 mRNA levels. (**B**) Representative pictures of Sirtuin 1 protein staining in the liver. Bars represent 10 µm. (**C**) Quantification of nuclear Sirtuin 1-positivity. (**D**) Representative Western blotting of Sirtuin 1 and its quantification compared with β-actin. (**E**) Mature miR-34a-5p levels. Values are mean ± SEM. * *p* < 0.05 vs. WT STD, § *p* < 0.05 vs. HET STD, ** *p* < 0.05 vs. WT HFD.

Because melatonin ameliorated dietary-induced NAFLD, we evaluated whether melatonin can affect the hepatic levels of miR-34a-5p which negatively controls SIRT1 expression mainly at the translational level [57]. First, we examined both SIRT1 mRNA and protein levels in all experimental groups. Intriguingly, the dietary challenge did not directly affect SIRT1 expression at the mRNA level (Figure 7A). However, SIRT1 protein decreased in the WT HFD group and was greatly enhanced in WT HFD mice drinking melatonin. Conversely, limited positive SIRT1 nuclei were detected in the HET STD mice, further reduced by HFD and not changed by melatonin intake (Figure 7B,C). These results were confirmed by Western blotting analysis in nuclear extracts (Figure 7D).

Interestingly, qPCR data revealed that hepatic miR-34a-5p levels increased in the WT HFD compared to the STD group. Remarkably, the treatment with melatonin significantly attenuated this increase, retrieving the miR-34a-5p expression at a basal level (Figure 7E). In HET mice, HFD determined a lesser, non-significant, increase of miR-34a-5p expression that was partially reduced by melatonin (Figure 7E). These data suggest that melatonin might restore SIRT1 by reducing mature miR-34a-5p levels in the liver of HFD-fed WT mice. However, the lack of up-regulation of SIRT1 after melatonin intake in HET mice and the modulation of miR-34a-5p in HET mice hinted at the presence of additional mechanisms maintaining SIRT1 expression at a low level in liver of HFD-fed HET mice despite melatonin intake.

## 4. Discussion

The current study indicated that oral melatonin (10 mg/kg), by influencing miR-34a/SIRT1/SREBP1 expression, ameliorated steatosis, ER stress, mitochondrial health and autophagy in high-fat diet-induced NAFLD in WT but not in HET mice. To the best of our knowledge, we firstly evidenced here a dual therapeutic melatonin role as a down-regulator of miR-34a-5p expression and as an up-regulator of SIRT1 expression in the liver. However, these crucial changes occurred in WT dietary obese mice. On the contrary, in HET mice only a trend of decrease in miR-34a-5p expression and no effect on SIRT1 protein were observed. A strict melatonin/SIRT1 crosstalk was reported to sustain crucial mitochondrial function in hepatic cells and to prevent fission in diabetic heart, but the role of miR-34a-5p was lacking [58,59]. Thus, our results are in line with a recent study that reported melatonin benefits in neonatal rat brain inflammation through an inverse miR-34a-SIRT1 relationship [60]. Moreover, this study could have translational value because the same dose of melatonin for 3 months limited NAFLD in other rodent models and also in patients [61,62,63].

Recent evidences indicate that melatonin is beneficial in liver diseases by coordinating the crosstalk between miRNAs and related targets [64]. Indeed, aberrant miR-34a-5p up-regulation is crucial in obesity, type 2 diabetes and NAFLD progression to carcinoma [65,66]. Conversely, the antisense silencing of miR-34a-5p in NAFLD in rodents improved steatosis via over-expression of SIRT1 [67]. Similarly, liver-specific knockout mice for transcription coactivator Crtc2, a regulator of hyperglycemia and lipid metabolism, reduced miR-34a-5p and enhanced SIRT1 expressions under a fatty dietary challenge [68]. Here, we proposed that melatonin might act as a sort of “negative regulator” of miR-34a-5p in dietary-stressed WT mice when an excessive caloric intake forced lipogenesis, and the main activities of the endoplasmic reticulum and mitochondria.

Lipid droplet deposition in the liver occurred by SREBP1c. Mice over-expressing SREBP1c developed a fatty liver even under a normocaloric diet [69]. Notably, our findings indicated that melatonin limited nuclear SREBP1 in WT HFD mice, resulting in lower lipid deposition and attenuated glycaemia. In contrast, aberrant SREBP1, micro-steatosis and glycaemia persisted in HET HFD mice despite melatonin intake. Interestingly, SREBP1c mRNA and expression strongly depended on melatonin and SIRT1 in the metabolic syndrome and SIRT1 deficient mice developed sustained hepatic steatosis and obesity [70,71]. Indeed, SIRT1 is a pleiotropic enzyme placed at the crossroads of many metabolic pathways, like AMPK signaling, dysfunctional in HET mice fed HFD [72].

Remarkably, our results indicated that melatonin attenuated mitochondrial alterations and ER stress only in WT mice. Notably, mitochondria dynamic and shape are strictly dependent on SIRT1 and NAD^+^ biorhythm in NAFLD mice models [42,73]. Moreover, a lard-based diet triggered ER stress, abnormal endoribonuclease activity and miR-34a in NAFLD mice as previously reported [74,75]. Non-parenchymal cells are deeply involved in NAFLD grading and inflammation [76,77]. Remarkably, fibrosis and iron-stained F4/80 positive macrophages, IL6, as a pro-inflammatory cytokine, persisted in HET HFD mice even after melatonin intake. Exacerbated inflammation and fibrosis agree with a previous molecular study [23].

Intriguingly, here, efficient basal autophagy flux was detected in starved WT mice, but not in HET mice. Inefficient autophagy has been found also in both mice fed with HFD, and melatonin recovered autophagy and triggered mitophagy in WT mice only. Novel evidence indicates that melatonin can be a strong modulator of autophagy and survival in diseases and activation of autophagy was an effective therapy in NAFLD and NASH [78,79,80]. According to our findings in WT mice, melatonin protected fatty liver, regulating mitophagy and p62 protein that is necessary to drive dysfunctional mitochondria to autophagosomes for dismantling [81,82]. An interesting recent study reported that SIRT1, by coupling with Mfn2, is able to maintain adaptive mitophagy [83]_._

Notably, our findings demonstrated that melatonin enhanced Mfn2 signal in the hepatocytes of WT HFD mice and probably triggered mitophagy. Hence, we hypothesize that one of the best effects of melatonin in dietary induced NAFLD is to provide mitochondrial stasis, based on SIRT1 presence and mitophagy, according to Yamada et al. [84].

There are some limitations to this study. First, we did not measure SIRT1 activity but it is limited in NAFLD in rodents [85]. Second, we did not analyze chaperone-mediated autophagy but a previous study on obese mice indicated that this mechanism is not involved [86]. Third, we cannot rule out that melatonin could regulate other miRNAs targeting SIRT1 (i.e., miR-181b) [87]. Indeed, in a recent review, it has been deeply discussed that melatonin regulated the expression of many miRNAs, including miR-24, miR-155 and miR-16 [64]. However, to date, there is no data about the mechanisms activated by melatonin on the transcriptional control of miR-34a-5p. Finally, we did not measure endogenous melatonin levels that may influence different responses to dietary treatment during nighttime. However, in the C57BL/6J strain, the plasmatic melatonin level is very limited and probably irrelevant [88].

## 5. Conclusions

**Figure 8:**
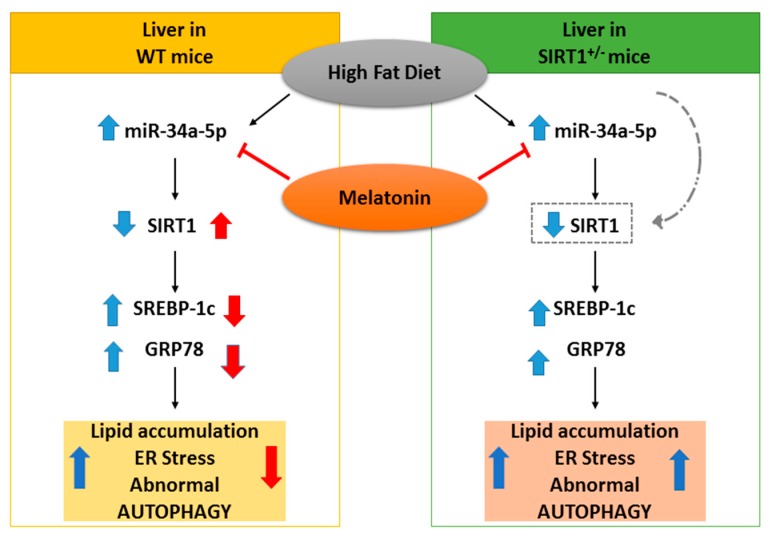
Hypothesized protective effect of melatonin in NAFLD mice. On the left, in WT mice, melatonin blocked the increase of miR-34a-5p, resulting in the up-regulation of its target SIRT1 and subsequently in the down-regulation adverse liver changes. On the right, in HET mice, melatonin decreased (as a trend) the expression of miR-34a-5p but was ineffective, due to lower SIRT1 levels. Blue and red arrows respectively represent high-fat diet and melatonin effects.

The current study indicates that heterozygous SIRT1 mice challenged by a hypercaloric lard-based diet developed severe oxidative mitochondrial damage, inflammation and fibrosis, all signs of advanced NAFLD staging. Recent study reported that metabolic stress affected endogenous SIRT1 activity necessary to mitigate mitochondrial stress [89]. Here, we showed that melatonin strongly decreases the hepatic miR-34a-5p expression levels and restores SIRT1 in dietary obese mice (Figure 8). Remarkably, the indole is unable to alleviate dietary induced NAFLD in mice expressing low SIRT1 enzyme. Even if our results are not conclusive, they provide suggestive evidence for a direct inhibitory role of melatonin on miR-34a-5p and adverse metabolic pathways in presence of full Sirtuin 1 protein availability. Another important point addressed here is that melatonin recovered autophagic flux and probably mitophagy, ameliorating mitochondrial damage. Finally, we evidenced that melatonin worked as a novel miR-34a-5p regulator and blocked NAFLD progression in mice. This last finding indicates that it might be promising to use dietary melatonin to prevent and treat obesity in patients.

## Supplementary Materials

The following are available online at https://www.mdpi.com/2073-4409/8/9/1053/s1, Figure S1: Indirect calorimetry data taken at the beginning, after seven or fifteen weeks of obesogenic diet plus melatonin. Figure S2: Lipid droplets stereology measured at ultrastructural level, Figure S3: Melatonin was ineffective in SIRT 1 heterozygous (HET) mice placed on a high-fat diet, Figure S4: WB analysis and relative quantification of p62 expression levels in wild type (WT) and in SIRT1 heterozygous mice fed standard (STD) and high fat (HFD) diet plus or not melatonin.
